# Integrated structural, computational, and genetic analyses link KASII-A^L263F^ to increased palmitic acid composition and highlight its potential in soybean oil breeding

**DOI:** 10.3389/fpls.2026.1983954

**Published:** 2026-10-12

**Authors:** Jiyoung Park, Kihwan Kim, Junseop Shin, Deokmoon Lee, Hyunsung Lee, Hyun Jo, Jong Tae Song, Won-Chan Kim, Jeong-Dong Lee

**Affiliations:** 1 Department of Applied Biosciences, Kyungpook National University, Daegu, Republic of Korea; 2 Upland Field Machinery Research Center, Kyungpook National University, Daegu, Republic of Korea; 3 Department of Integrative Biology, Kyungpook National University, Daegu, Republic of Korea

**Keywords:** AlphaFold2, EMS-induced mutant, KASII-A, palmitic acid, Pungsannamul, Seonpung, soybean

## Abstract

Soybean seed oil quality is governed by the relative proportions of five major fatty acids. Most genetic studies of soybean fatty acid mutants have evaluated allelic effects primarily on fatty acid composition, rather than quantifying their corresponding contents. Because fatty acid composition is expressed as relative percentage of total fatty acids, an increase in the proportion of one fatty acid is necessarily accompanied by a relative decrease in others, making it difficult to distinguish compositional shifts from changes in the actual amount accumulated per unit seed mass. In this study, we characterized PE1604, an EMS-induced mutant derived from the Korean soybean cultivar Pungsannamul and evaluated fatty acid phenotypes using both composition and contents. Sequence analysis identified a C to T transition in *KASII*-*A* (Glyma.17G047000) that resulted in an L263F amino acid substitution at a leucine residue conserved among KASII-A homologs from 11 species of the family Fabaceae. AlphaFold2 models superposed on the *Escherichia coli* (*E*. *coli*) FabF complex with *crypto* - 1b-AcpP (C16-AcpP) placed Leu263 within the buried hydrophobic core of KASII-A, distant from the catalytic residues Cys240, H380, and H416 in the active site. FoldX predicted a destabilizing effect of 6.83 kcal/mol for L263F, attributed to van der Waals clashes. Rosetta analysis also indicated a destabilizing energetic change of 11.57 REU. In F_2_ populations derived from Pungsannamul × PE1604 and Seonpung × PE1604, palmitic acid composition increased from 12.1% in *KASII*-*A* homozygotes to 18.1% in *kasII*-*a* homozygotes and 11.7% in *KASII*-*A* homozygotes to 17.4% in *kasII*-*a* homozygotes, respectively. In addition, analysis of fatty acid content showed that palmitic acid content in *kasII*-*a* homozygotes progeny paralleled the difference in palmitic acid content between the parental lines. Seonpung contained approximately 33.5 mg/g seed weight palmitic acid content, and *kasII*-*a* homozygotes progeny from the Seonpung × PE1604 cross accumulated 51.9 mg/g seed weight, wherease Pungsannamul conta0ined approximately 23.1 mg/g seed weight and the corresponding progeny from the Pungsannamul × PE1604 cross accumulated 38.2 mg/g seed weight. These results suggest that the L263F amino acid substitution may affect KASII-A through altered local packing and protein stability rather than direct perturbation of the catalytic site.

## 1 Introduction

Soybean (*Glycine max* [L.] Merr.) is the most important oilseed crop worldwide and supplies a large share of the vegetable oil and plant protein globally (Singh et al., 2008; Liu et al., 2020). The mature seed contains approximately 20% oil and 40% protein, and both traits are influenced by genotype and by environment (Clemente and Cahoon, 2009; Min et al., 2015). Soybean oil is used in foods, in animal feed, and in industrial applications including biodiesel (Lu et al., 2011; Sieverding et al., 2015; White et al., 2017; Kafer et al., 2026). Five fatty acids account for almost all of the oil, namely palmitic acid (C16:0) and stearic acid (C18:0), oleic acid (C18:1), linoleic acid (C18:2), and linolenic acid (C18:3). Conventional soybean cultivars produce oil containing approximately 12% palmitic acid, 4% stearic acid, 23% oleic acid, 53% linoleic acid, and 8% linolenic acid (Clemente and Cahoon, 2009; Hudson, 2012). Total fatty acid composition and content are themselves under polygenic control, and considerable effort has been directed at identifying favorable haplotypes and at balancing oil against protein in elite germplasm (He et al., 2024; Shi et al., 2025; Bu et al., 2026). Among them, palmitic acid is interest as a breeding target. Soybean oil enriched in saturated fatty acids, including palmitic acid or stearic acid, enhances oxidative stability and provides solid-fat functionality without the need for partial hydrogenation, which generates *trans*-fatty acids, facilitating the production of *trans*-free margarine (Kok et al., 1999; Neff and List, 1999; Ascherio and Willett, 1997; Bihola et al., 2025).

In plants, fatty acid synthesis occurs primarily in plastids, where acyl chains are carried by acyl carrier protein (ACP) and elongated through a series of enzyme-catalyzed reactions (Thelen and Ohlrogge, 2002; Guschina et al., 2014). Fatty acid chains are extended through successive two-carbon additions from malonyl-ACP, with 3-ketoacyl-ACP synthases catalyzing the decarboxylative condensation step of each elongation cycle. Among these enzymes, KASII catalyzes the condensation of palmitoyl (C16:0)-ACP with malonyl-ACP to form 3-ketostearoyl-ACP (Guschina et al., 2014). Subsequent reduction, dehydration, and reduction reactions generate stearoyl (C18:0)-ACP, which can be desaturated to oleoyl (C18:1)-ACP by stearoyl-ACP desaturase (Shanklin and Somerville, 1991; Pidkowich et al., 2007). Therefore, KASII catalyzes a key step in C16-to-C18 fatty acid elongation, and reduced KASII activity limits the conversion of palmitoyl-ACP to stearoyl-ACP, resulting in an increased proportion of palmitic acid in seed oil (Pidkowich et al., 2007). Soybean contains two homologous *KASII* genes, *KASII*-*A* (Glyma.17G047000) and *KASII*-*B* (Glyma.13G112700). Mutations in *KASII*-*A* generally produce more increases in seed palmitic acid than mutations in *KASII*-*B* (Aghoram et al., 2006; Anai et al., 2012; Devereaux et al., 2024). Multiple induced and naturally occurring alleles of these genes have been identified in mutant populations and soybean germplasm, including premature stop codons, splice-site mutations, deletions, and missense substitutions at conserved amino acid residues (De Vries et al., 2011; Head et al., 2012; Hudson, 2012; Hudson and Hudson, 2021; Derbyshire et al., 2023; Kim et al., 2025). These alleles establish *KASII* as a major genetic determinant of seed palmitic acid composition and provide useful genetic variation for modifying the saturated fatty acid in soybean oil.

However, genetic studies of soybean seed fatty acid have predominantly characterized mutant phenotypes on the basis of fatty acid composition, with individual fatty acids expressed as percentages of total fatty acids (Aghoram et al., 2006; De Vries et al., 2011; Anai et al., 2012; Head et al., 2012; Devereaux et al., 2024; Kim et al., 2025). While analysis of soybean seeds fatty acid composition effectively identifies changes in relative fatty acid profiles, it does not determine whether the content of a specific fatty acid per unit seed mass has changed. The content of an individual fatty acid depends on both its relative proportion and the total amount of fatty acids accumulated in the seed. Because seed oil content is itself a genetically variable trait and a major target of soybean improvement (He et al., 2024; Shi et al., 2025; Bu et al., 2026), similar changes in fatty acid composition may result in different amounts of a target fatty acid among cultivars. Therefore, the soybean breeding value of a fatty acid modifying allele should be evaluated in terms of its effects on fatty acid composition and content per unit seed mass, together with the consistency of these effects across different cultivars. An allele that acts on the composition of seed oil is not, by itself, expected to account for variation in total fatty acid accumulation among cultivars, and the two descriptions are complementary rather than alternatives.

In this study, we characterized PE1604, an ethyl methanesulfonate (EMS)-induced mutant derived from the Korean soybean cultivar Pungsannamul that exhibits an elevated palmitic acid composition. Sequence analysis of *KASII*-*A* and *KASII*-*B* identified a novel L263F amino acid substitution in KASII-A. To assess the structural consequences of this substitution, we combined protein structure prediction, structural comparison with the *Escherichia coli* (*E*. *coli*) FabF-C16-AcpP complex, and computational mutagenesis using two independent energy functions to determine the location of Leu263 relative to the active site region and to evaluate its predicted effects on protein stability. In addition, we investigated the phenotypic effect of the *KASII*-*A* allele in F_2_ populations derived from crosses involving two different cultivars. Fatty acid composition and content were quantified in the same F_2_ seeds to determine whether the effect of *KASII*-*A* allele on palmitic acid composition was reproducible across F_2_ populations and how differences in fatty acid accumulation influenced the palmitic acid content associated with the *KASII*-*A* allele. Together, these analyses were designed to define the structural basis of the KASII-A^L263F^ variant and to evaluate its potential utility for modifying palmitic acid composition and content in soybean breeding.

## 2 Materials and methods

### 2.1 Plant materials and population development

The mutant line PE1604 was selected from EMS-induced mutant population developed in the Korean soybean cultivar Pungsannamul (Chae et al., 2013). Among 163 EMS-induced mutant lines, PE1604 was identified as a high palmitic acid mutant based on its elevated seed palmitic acid composition of 16.14% (**Supplementary Table 1**). To investigate the inheritance of mutant allele from PE1604, two different cross populations were developed using PE1604 and two Korean soybean cultivars Pungsannamul and Seonpung (Lee et al., 2015; Kim et al., 2017). Pungsannamul and Seonpung were observed to have normal palmitic acid composition (**Supplementary Table 1**).

The initial crosses were performed during the summer of 2023 at the field at the affiliated experiment and practice fields of Kyungpook National University (Gunwi, 36°07′N, 128°38′E, Republic of Korea) to produce F_1_ seeds. F_1_ plants were grown during the winter of 2023 to 2024 in a greenhouse of Kyungpook National University (Daegu, 35°53′N, 128°36′E, Republic of Korea). The photoperiod was extended to 12 h using supplemental lighting of approximately 54 µmol/m^2^/s and the minimum temperature maintained above 20°C. Each individual F_2_ seed was processed separately to allow direct matching of the *KASII*-*A* genotype with the fatty acid phenotype of the same seed. Each population comprised 150 individual F_2_ seeds.

### 2.2 DNA extraction, amplification, and sequencing

The sequence of the *KASII*-*A* gene and *KASII*-*B* were retrieved from Phytozome database (https://phytozome-next.jgi.doe.gov/, Wm82.a4.v1). Five primer pairs were designed for each gene to generate overlapping PCR amplicons covering the genomic region, including all exons and introns (**Supplementary Table 2**). For each F_2_ individual, a single F_2_ seed was ground separately into a fine powder. The resulting seed powder was divided into separate portions for genomic DNA (gDNA) extraction and fatty acid analysis, thereby allowing genotypic and phenotypic data to be obtained from the same individual seed. For gDNA extraction, an aliquot of the seed powder was transferred to a microcentrifuge tube and mixed with 300 µl of modified Edwards extraction solution (400 mM LiCl, 200 mM Tris-HCl, 25 mM EDTA, and 1% (v/v) SDS, pH 9.0). The gDNA preparation procedure according to the modified Edwards extraction procedure described previously (Kim et al., 2023a). Polymerase chain reaction (PCR) was performed in 50 µL reaction in composition of 1 µL of template DNA (30 ng/µL), 0.5 µM of forward and 0.5 µM of reverse primers, 10 µL of 5× PrimeSTAR Buffer, 10 mM of dNTP, and 0.5 µL of PrimeSTAR HS DNA Polymerase (Takara Bio Inc, Japan). The PCR reaction conditions were as follows: initial denaturation at 98°C for 5 min; followed by 40 cycles of denaturation at 98°C for 30 s, annealing at 58°C for 15 s, and elongation at 72°C for 2 min; then extension step at 72°C for 5 min. PCR amplicons were purified using EXprep™ Gel SV kit (GeneAll, Republic of Korea).

To verify the nucleotide sequences, the purified PCR products were cloned into the pTOP Blunt V2 vector using TOPcloner TA-Blunt kit (Enzynomics, Republic of Korea) and transformed into *E*. *coli* DH5α (Kim et al., 2023b). Transformed *E*. *coli* cells were spread onto LB agar plates containing 1.5% (w/v) agar, 50 µg/L kanamycin, and 100 µg/L ampicillin. Colonies growing on the selective plates were picked and inoculated into liquid LB medium containing the same antibiotics, followed by overnight incubation. Plasmid DNA was isolated from the bacterial culture using Exprep™ Plasmid SV mini kit (GeneAll, Republic of Korea). The nucleotide sequences of the cloned PCR fragments were determined by Sanger sequencing.

### 2.3 Sequence alignment and phylogenetic analysis

The amino acid sequence of KASII-A homologs from 11 species of the family Fabaceae were retrieved from the National Center for Biotechnology Information (NCBI, https://www.ncbi.nlm.nih.gov). Accessions used were *Arachis duranensis* (XP_020998676.1), *Arachis hypogaea* (KAL4397468.1), *Cajanus cajan* (XP_020226547.1), *Flemingia macrophylla* (KAL2338810.1), *Glycine soja* (XP_028210164.1), *Phaseolus vulgaris* (XP_068463727.1), *Psophocarpus tetragonolobus* (KAK7388293.1), *Sphenostylis stenocarpa* (CAJ1940558.1), *Vigna mungo* (WVZ11225.1), *Vigna radiata* (XP_014509299.1), and *Vigna unguiculata* (XP_027920046.1). Multiple sequence alignment was performed with ClustalW (BioEdit software). A phylogenetic tree was constructed in MEGA 11 by the neighbor-joining method with 1, 000 bootstrap replicates (Tamura et al., 2021).

### 2.4 Protein structure prediction

Three-dimensional structure of KASII-A^L263F^ amino acid substitution from PE1604 were predicted with AlphaFold2 in ColabFold using default settings and without structural templates (Jumper et al., 2021; Mirdita et al., 2022; Steinegger and Söding, 2017). The predicted structure of wild-type KASII-A from Pungsannamul was obtained from our previous study (Kim et al., 2025) and used for structural comparison with KASII-A^L263F^. Model quality was assessed using the predicted local-Distance Difference Test (plDDT) score and Predicted Aligned Error (PAE), which provide estimates of local residue-level confidence and confidence in the relative positioning of residues, respectively (Mariani et al., 2013). The predicted KASII-A structures were superimposed onto the cross-linked crystal structure of *E. coli* FabF in complex with C16-AcpP (PDB accession no. 6OKG) to examine the position of the substituted residue relative to the ACP-binding interface, catalytic residues, and acyl-binding region. FabF is the bacterial KASII homolog and catalyzes the elongation of palmitoyl-ACP substrates (Mindrebo et al., 2020). The Multiple Alignment of Protein Structure (MAPS) was performed using default options for the input parameters. All illustrations of the protein structures of KASII-A and its homologs were generated using PyMOL v.3.0.

### 2.5 Computational analysis of the L263F amino acid substitution on protein stability

The FoldX was used to evaluate the effect of the L263F amino acid substitution on KASII-A protein stability using the empirical energy function (Guerois et al., 2002; Schymkowitz et al., 2005). The predicted structure of KASII-A from Pungsannamul was used as the starting model and was first processed with the RepairPDB command to relieve local interactions. The L263F amino acid substitution was introduced with the BuildModel command using five independent runs. Changes in folding free energy (ΔΔG) were calculated in kcal/mol, with positive values indicating destabilization relative to the wild-type KASII-A protein structure. Energy terms were decomposed using the output of the BuildModel difference file, and per residue contributions were extracted from SequenceDetail output. Saturation mutagenesis at Gly156, Leu160, Leu227, Trp229, and Leu263 was performed by substituting each position with all 19 alternative amino acids. Independent stability predictions were obtained with Rosetta through the ROSIE2 point mutation stabilization application (https://r2.graylab.jhu.edu), which applies the Rosetta all atom energy function within the Rosetta modelling framework (Thieker et al., 2022; Alford et al., 2017; Koehler Leman et al., 2020). Rosetta energies are reported in Rosetta Energy Units (REU). Because the two force fields are parameterized on different reference data and are not expressed on a common scale, FoldX and Rosetta results were compared by rank correlations rather than by absolute value. The choice of these two methods follows a systematic benchmark in which FoldX and Rosetta showed the strongest correspondence with mutant fitness measured by deep mutational scanning, and in which a consensus of the two performed better than either alone (Gerasimavicius et al., 2023).

### 2.6 Fatty acid methyl esters analysis

Fatty acid composition and content were determined by Agilent 7890A Gas Chromatography with flame ionization detection (GC-FID) and DB-FFAP capillary column (30 m in length × 0.25 mm in diameter × 0.25 µm in thickness of film; Agilent Technologies Inc, USA). Soybean oil was extracted from ground seed in 1 mL of extraction solution from a second aliquot of the powder of the same individual seed sued for gDNA extraction (chloroform: hexane: methanol [8:5:2, v/v/v]) for 12 h at room temperature. The 200 µL supernatants of the soybean oil were mixed with 75 µL of methylation reagent (0.25 M methanolic sodium methoxide: petroleum ether: ethyl ether [1:5:2, v/v/v]). The fatty acid methyl esters were extracted with 725 µL of hexane. The hexane layer was analyzed using an Agilent 7890A GC-FID. A 2 µL sample was injected for analysis. Nitrogen (N_2_) was used as the carrier gas at a flow rate of 1.5 mL/min. The injector temperature was maintained at 230 °C with a split ratio of 50:1. The oven temperature was set at 210 °C, and the detector temperature was maintained at 250 °C. Fatty acid methyl esters were characterized by a comparison of retention time with a FAME standard mixture (FAME #16, Restek).

Fatty acids were identified by comparison of retention times with a 3% (v/v) FAME #16 standard mixture (**Supplementary Figure 1A**). For quantification, the FAME # 16 standard mixture was serially diluted and analyzed under same GC-FID conditions. Calibration curves for the five major fatty acids were generated by plotting the peak area of each fatty acid against its known concentration in the FAME #16 standard mixture (**Supplementary Figures 1B, C**). Each calibration level was analyzed with five replicate injections, and a linear regression equation and coefficient of determination (R^2^) were calculated for each fatty acid. Fatty acid composition was calculated as the proportion of each of the five major fatty acids (palmitic acid, stearic acid, oleic acid, linoleic acid, and linolenic acid) relative to their summed amount and was expressed as a percentage. Fatty acid content (*A_FA_*) was calculated on a seed mass basis using the following equation:


$$A_{FA}(mg/g\;seed\;weight)=\frac{C_{GC}(\text{m}g/mL)\times DF\times V_{final}\;(mL)}{f_{aliquot}\times m_{analyzed\;seed\;weight}\;(g)}$$

Where *A_FA_* is the fatty acid content expressed as mg FA/g seed weight, *C_GC_* is the concentration of the individual fatty acid (mg/mL) calculated by applying the GC peak area to the corresponding linear regression equation obtained from the FAME #16 standard mixture calibration curve (**Supplementary Figure 1C**). *DF* is the final dilution factor, *V_final_* is the final reconstitution volume after derivatization (mL), *f_aliquot_* is the fraction of the total extract used for derivatization, and *m_analyzed seed weight_* is the mass of the seed sample used for extraction (g).

### 2.7 Allele-specific molecular marker development and genotyping of segregating populations

A SimpleProbe genotyping assay for the *KASII*-*A* allele was developed to detect the lines carrying the identified mutation based on the disassociation kinetics of probe oligonucleotides. Asymmetric amplification was performed in 20 µL reactions containing 0.5 µM forward primer, 0.2 µM reverse primer, and the 2 µM of allele specific probe, 1× reaction buffer [40 mM Tricine-KOH, 16 mM KCl, 3.5 mM MgCl_2_, 3.75 µg/mL bovine serum albumin (BSA), and 10 mM dNTPs, pH 8.0], 5% (v/v) DMSO, and 0.2× Titanium Taq polymerase (BD Biosciences, CA). The SimpleProbe genotyping assay was performed in a 96-well plate using the LightCycler 480 II real-time PCR instrument (Roche Applied Sciences, Germany). The PCR conditions were as follows: initial denaturation at 95°C for 5 min; followed by 50 cycles of denaturation at 95°C for 30 s, annealing at 55°C for 30 s, and elongation at 72°C for 30 s; then extension step at 72°C for 5 min, after which a melting curve was acquired from 54°C to 72°C. The fluorescence was read every 0.1°C within 54°C to 72°C. Genotypes were called from melting peak positions, and representative melting profiles together with probe information and primer sequences are shown in **Supplementary Figure 2**.

F_2_ seeds were genotyped for KASII-A using a SimpleProbe assay on LightCycler 480 II instrument. The single-nucleotide match or mismatch was determined using an integration of the fluorescence disappearance and increasing melting temperature. The homozygous wild-type *KASII-A* was detected at approximately 67 °C, indicating a peak: the homozygous *kasII-a* showed a peak at approximately 59 °C, while heterozygous alleles produced both peaks.

### 2.8 Statistical analysis

All experiments were tested by one-way analysis of variance (ANOVA) followed by Tukey’s honestly significant difference (HSD) test (*p* < 0.05) using SAS v9.4 (SAS Institute Inc., USA). Goodness of fit of observed genotypic segregation to the expected Mendelian ratio was tested by chi-square analysis from each segregation population. Frequency distributions of F_2_ phenotypes were plotted as histograms with bin widths selected by Scott rule.

## 3 Results

### 3.1 A novel KASII-A allele encoding a L263F amino acid substitution in PE1604

PE1604 was identified within an EMS-induced mutant population of Pungsannamul as a line with markedly elevated palmitic acid composition (**Supplementary Figure 3**). Because KASII catalyzes the elongation of palmitoyl-ACP to stearoyl-ACP and mutations in soybean *KASII*-*A* and *KASII*-*B* are known to increase seed palmitic acid (Aghoram et al., 2006; De Vries et al., 2011; Anai et al., 2012; Head et al., 2012; Hudson, 2012; Hudson and Hudson, 2021; Derbyshire et al., 2023; Devereaux et al., 2024; Kim et al., 2025), these two genes were selected as primary candidates underlying the PE1604 phenotype. To determine the genetic basis of this phenotype, the genomic regions of *KASII*-*A* and *KASII*-*B* were amplified in five overlapping fragments each and sequenced in PE1604 (**Supplementary Figure 4**, **5**). No polymorphism affecting the coding sequence was detected in *KASII*-*B*, although a single Thymine (T) insertion was identified in 1^st^ intron (**Supplementary Figure 5**). However, a single nucleotide substitution from Cytosine (C) to T was identified at coding sequence position 787^th^ of the 6^th^ exon in the coding region of the *KASII*-*A* gene of PE1604 (**Figures 1A, B**; **Supplementary Figure 4**). The PE1604 carried single-nucleotide polymorphism (SNP) mutations which resulted in missense amino acid change from Leucine (Leu, L) to Phenylalanine (Phe, F) at amino acid position 263 (denoted and used from here on as L263F) (**Supplementary Figure 6**). Alignment of KASII-A amino acid sequences from Pungsannamul and PE1604 with KASII homologs from 11 species of the family Fabaceae showed that Leu263 was invariant across all sequences examined (**Figure 1C**; **Supplementary Figure 7A**). Phylogenetic analysis placed the KASII-A amino acid sequence from Pungsannamul and PE1604 within the expected clade relative to the other legume homologs (**Supplementary Figure 7B**). The conservation of Leu263 among KASII homologs across the family Fabaceae suggests structural or functional constraint at this position. Although leucine and phenylalanine share hydrophobic properties, their side chains differ substantially in chemical structure. Leucine contains a branched aliphatic side chain, whereas phenylalanine contains a bulkier aromatic side chain, suggesting that the L263F amino acid substitution may affect protein stability. Given the conservation of Leu263, we investigated the structural consequences of the KASII-A^L263F^ amino acid substitution in PE1604 and whether it affect the local packing environment of KASII-A. Structures of KASII-A from Pungsannamul (hereafter KASII-A) and KASII-A^L263F^ from PE1604 (hereafter KASII-A^L263F^) were predicted with AlphaFold2 (**Figure 1D**). The plDDT and PAE scores revealed that except for unmodelled N-terminal region containing the initial 78 residues, the protein structure is of high confidence and suitable for any application that benefits from high accuracy (**Supplementary Figure 8**). Superposition of the two models revealed no observable change in the overall protein fold, whereas structural differences were localized around residue 263. In KASII-A from Pungsannamul, Leu263 formed close contacts with Gly156, Leu160, Leu227, and Trp229, with interatomic distances ranging from 3.8 to 4.2 Å. In KASII-A^L263F^ from PE1604, the bulkier aromatic ring of Phe263 altered these local contacts, with interatomic distances becoming shorter and others longer than those in the KASII-A from Pungsannamul. These results suggest a local rearrangement of residue packing around position 263, while the overall protein structure of KASII-A remained unchanged.

**Figure 1:**
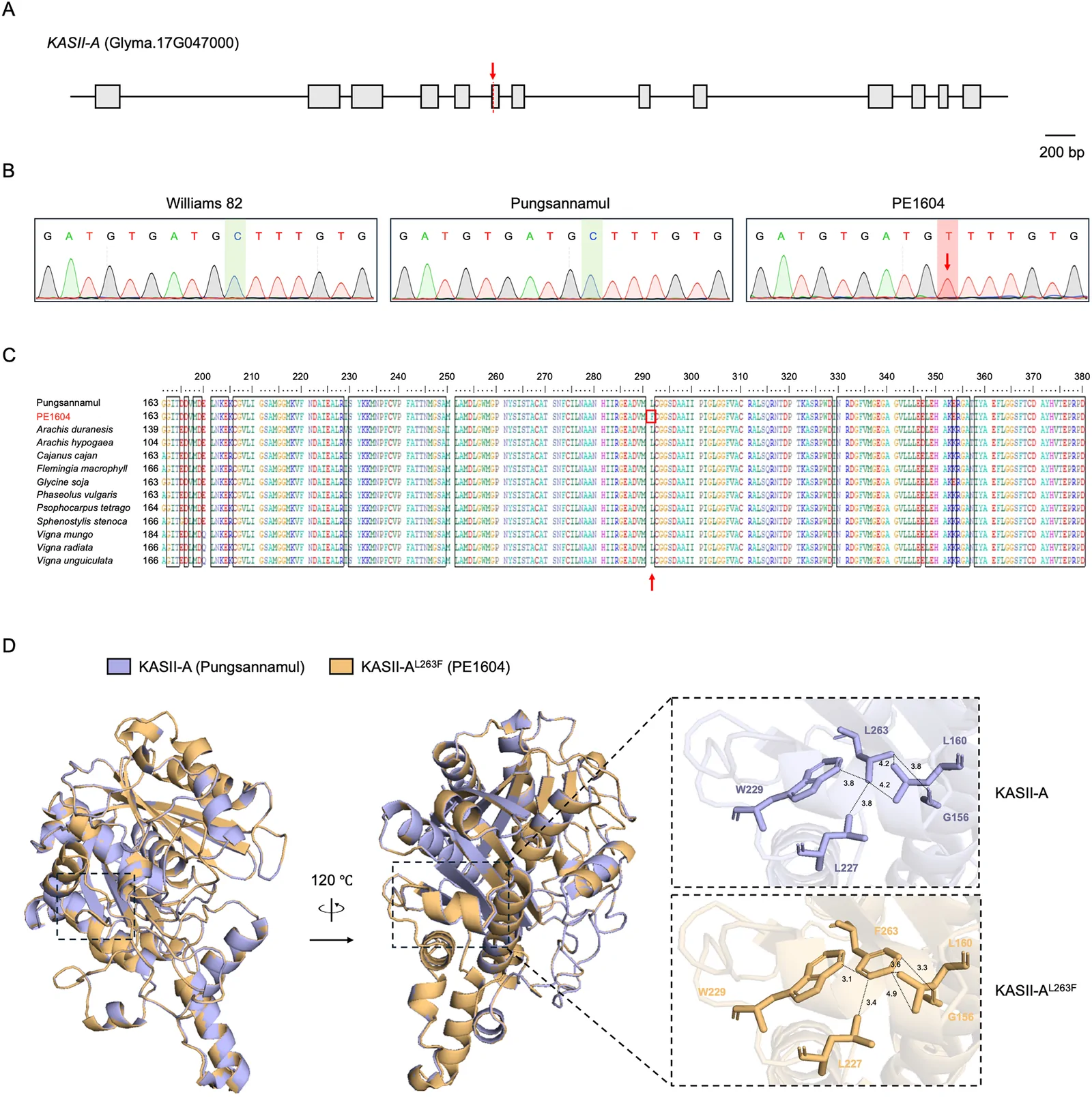
Molecular characterization and structural analysis of KASII-A^L263F^ in PE1604. **(A)** Gene structure of *KASII-A* (Glyma.17G047000). Exons are represented by boxes and introns by lines. The red arrow indicates the position of the mutation associated with the L263F amino acid substitution in PE1604. Scale bar, 200 bp. **(B)** Sanger sequencing chromatograms of *KASII-A* from Williams 82, Pungsannamul, and PE1604. The red-highlighted position indicates the C-to-T nucleotide substitution in PE1604, resulting in the L263F amino acid substitution. **(C)** Multiple sequence alignment of KASII-A amino acid sequences from Pungsannamul and PE1604 with KASII-A homologs from 11 species of the family Fabaceae. The red box and arrow indicate the amino acid position of the L263F substitution in PE1604. **(D)** Superposition of the three-dimensional structures of KASII-A from Pungsannamul and KASII-A^L263F^ from PE1604. Enlarged views show the local structural environments surrounding L263 in Pungsannamul and F263 in PE1604. Numbers indicate inter-residue distances in Å.

### 3.2 KASII-A^L263F^ is structurally distinct from the KASII-A active site

KASII catalyzes the condensation of palmitoyl-ACP with malonyl-ACP to form 3-ketostearoyl-ACP, which is subsequently reduced, dehydrated, and reduced again to generate stearoyl-ACP (**Figure 2A**). The KASII condensation reaction requires ACP to dock onto the KASII surface and deliver the ACP-bound acyl chain into the active-site channel toward the catalytic residue (Rossini et al., 2018; Mindrebo et al., 2020). We hypothesize that the high palmitic acid composition of PE1604 is associated with reduced KASII-A activity. To investigate whether Leu263 is positioned to directly affect the catalytic residues (Cys240, His380, and His416) within the KASII-A active site, we used the crystal structure of *E*. *coli* FabF in complex with *crypto* - 1b-AcpP (C16-AcpP) (PDB accession no. 6OKG), in which the C16-α-bromopantetheineamide probe (1b) extends from AcpP into the FabF active site (Mindrebo et al., 2020). The acyl substrate is carried by the 4’-phosphopantetheine prosthetic group, which provides a flexible arm terminating in a thiol group (-SH) that forms a thioester linkage with the acyl chain (**Figures 2B, C**). The 4’-phosphopantethein group is covalently bound to Ser36 of ACP through a phosphodiester bond (**Figure 2D**), which allows the docked acyl substrate to reach the active site (Sulpizio et al., 2021; Yeon et al., 2022). Therefore, *E*. *coli* FabF in complex with C16-AcpP provide a structural reference for locating the corresponding substrate-binding and catalytic residues in KASII-A. Superposition of the predicted KASII-A protein structure from Pungsannamul and KASII-A^L263F^ from PE1604 onto the FabF-C16-AcpP complex showed that residue 263 is spatially separated from the catalytic residues within the KASII-A active site (**Figure 2E**). Residue 263 was positioned away from the path occupied by the C16 substrate mimic within the acyl-binding channel. In addition, the relative arrangement of the catalytic residues and the overall protein structure of the active site were essentially unchanged between KASII-A from Pungsannamul and KASII-A^L263F^ from PE1604. These results indicate that the L263F amino acid substitution is unlikely to reduce KASII-A activity through direct alteration of the catalytic residues or substrate-binding channel, suggesting that its effect arises from structural changes outside the active site.

**Figure 2:**
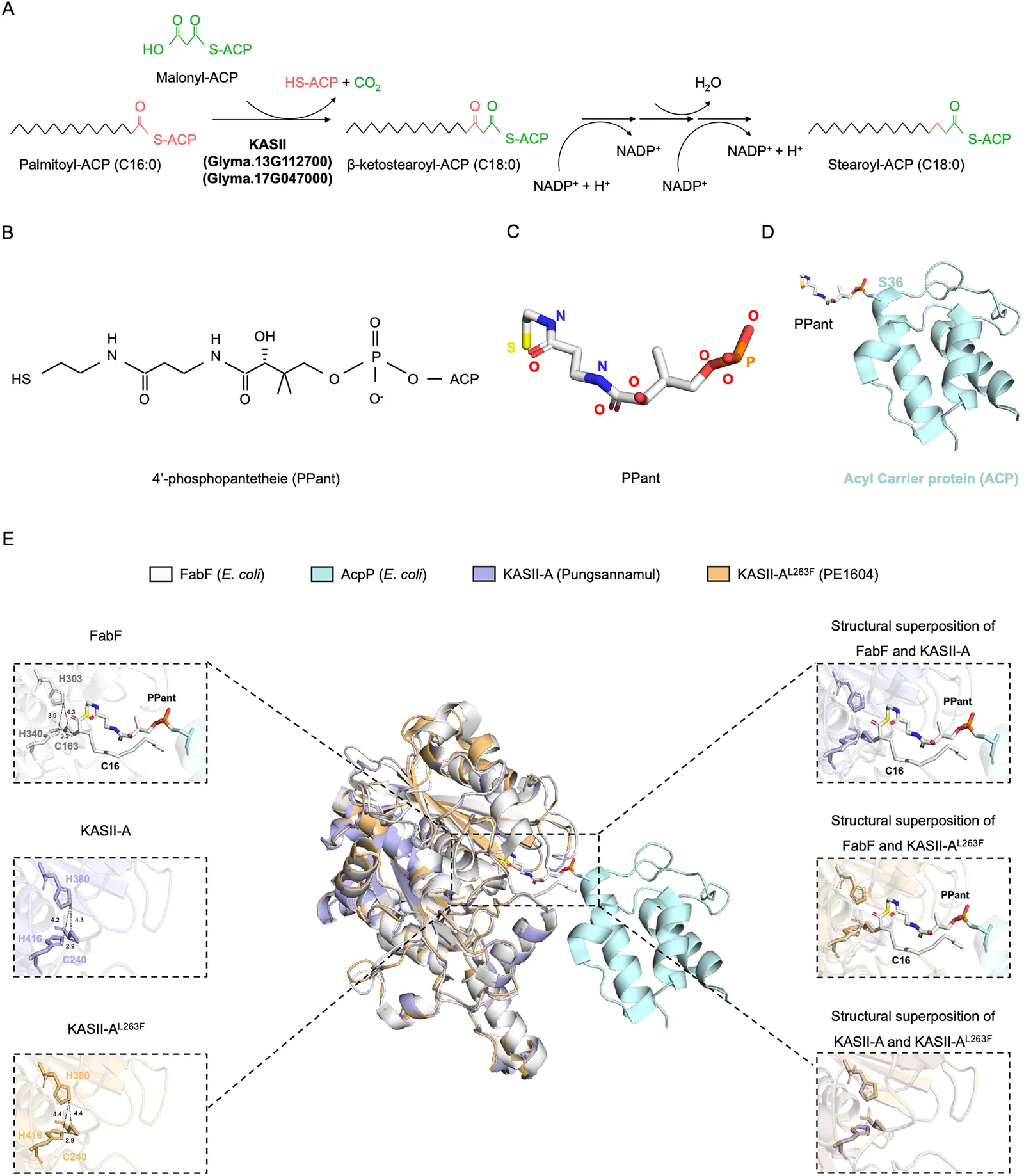
Structural analysis of KASII-A and KASII-A^L263F^ based on the structure of the *E*. *coli* FabF complex with C16-AcpP. **(A)** Schematic representation of KASII-mediated fatty acid elongation pathway. KASII catalyzes the condensation of palmitoyl (C16:0)-ACP with malonyl-ACP to form β-ketostearoyl-ACP, which is subsequently converted to stearoyl (C18:0)-ACP. **(B)** Chemical structure of 4’-phosphopantetheine (PPant), the prosthetic group of acyl carrier protein (ACP). **(C)** Three-dimensional representation of PPant. **(D)** Structural representation of ACP showing PPant covalently attached to Ser36 (S36) of ACP. **(E)** Structural comparison of KASII-A and KASII-A^L263F^ based on the structure of the *E*. *coli* FabF complex with C16-AcpP (PDB accession no. 6OKG). The predicted structures of KASII-A and KASII-A^L263F^ were superimposed onto the *E*. *coli* FabF structure to compare their substrate-binding environments. The structures of *E*. *coli* FabF, its AcpP, KASII-A, and KASII-A^L263F^ are shown in white, cyan, navy, and orange, respectively. Each molecule is identified in the figure by its protein name, with the source organism or soybean line given in parenthese. Enlarged views show the spatial arrangement of residues surrounding the Palmitoyl acyl chain and PPant. Pairwise structural superpositions of *E*. *coli* FabF and KASII-A, of *E*. *coli* FabF and KASII-A^L263F^, and of KASII-A and KASII-A^L263F^ are shown on the right. Numbers indicate inter-residue distances in Å.

### 3.3 Computational analyses predict destabilization of KASII-A^L263F^

To quantify the energetic effects of the L263F amino acid substitution, computational analyses were performed using FoldX and Rosetta. Gly156, Leu160, Leu227, and Trp229 were identified as residues in close spatial proximity to residue 263 and were investigated together with Leu263. Gly156 and Leu160 are located within the α3-helix, Leu227 within the α8-helix, and Leu263 or Phe263 within the β3-sheet, whereas Trp229 is located in a loop region (**Figures 3A, B**). Sequence alignment of KASII-A homologs from 11 species of the family Fabaceae showed these residues are located within highly conserved regions. Leu263 and four residues located in close spatial proximity to Leu263 (Gly156, Leu160, Leu227, and Trp229) were systematically substituted with all 19 alternative amino acids to evaluate the relationship between side chain properties and energetic effects (**Figures 3C, D**). FoldX predicted that the L263F amino acid substitution destabilizes KASII-A by 6.83 kcal/mol, while Rosetta independently predicted an unfavorable energy change of 11.57 REU. In addition, four residues located in close spatial proximity to Leu263 showed that the energetic penalty at each position was generally minimized for substitutions with side chain volumes similar to that of the wild-type residue. At Gly156, which lacks a side chain except a hydrogen atom, the energetic penalty increased as the volume of the substituted residue increased. In contrast, at Trp229, which has the largest side chain among the residues investigated, substitutions with smaller residues were increasingly unfavorable. At Leu263, substitutions with either smaller or larger side chains than leucine generally increased the energetic penalty. This pattern indicates that the side chain volume at residue 263 is closely matched to its local structural environment. Energy decomposition of the FoldX calculation showed that approximately 78% of the total unfavorable energy change associated with the L263F amino acid substitution was attributable to the van der Waals clash term, with the remaining contribution distributed among solvation and backbone-related terms (**Figure 3E**). Analysis of the energy contribution of individual residues within KASII-A^L263F^ showed the effect of L263F amino acid substitution was not confined to residue 263 but was distributed across residues surrounding the substitution site (**Figure 3F**). Leu160 showed the largest unfavorable energy contribution, accounting for 1.92 kcal/mol, of which 1.68 kcal/mol originated from van der Waals clashes. Consistent with this energetic effect, the minimum distance between Leu160 and residue 263 decreased from 4.2 Å in wild-type KASII-A containing Leu263 to 3.6 Å in KASII-A^L263F^ containing Phe263, indicating tighter local packing around the substituted residue (**Figure 1D**; **Figure 3B**). Taken together, the structural and computational analyses predict that the L263F amino acid substitution destabilizes KASII-A through unfavorable local packing rather than through direct alteration of the active site.

**Figure 3:**
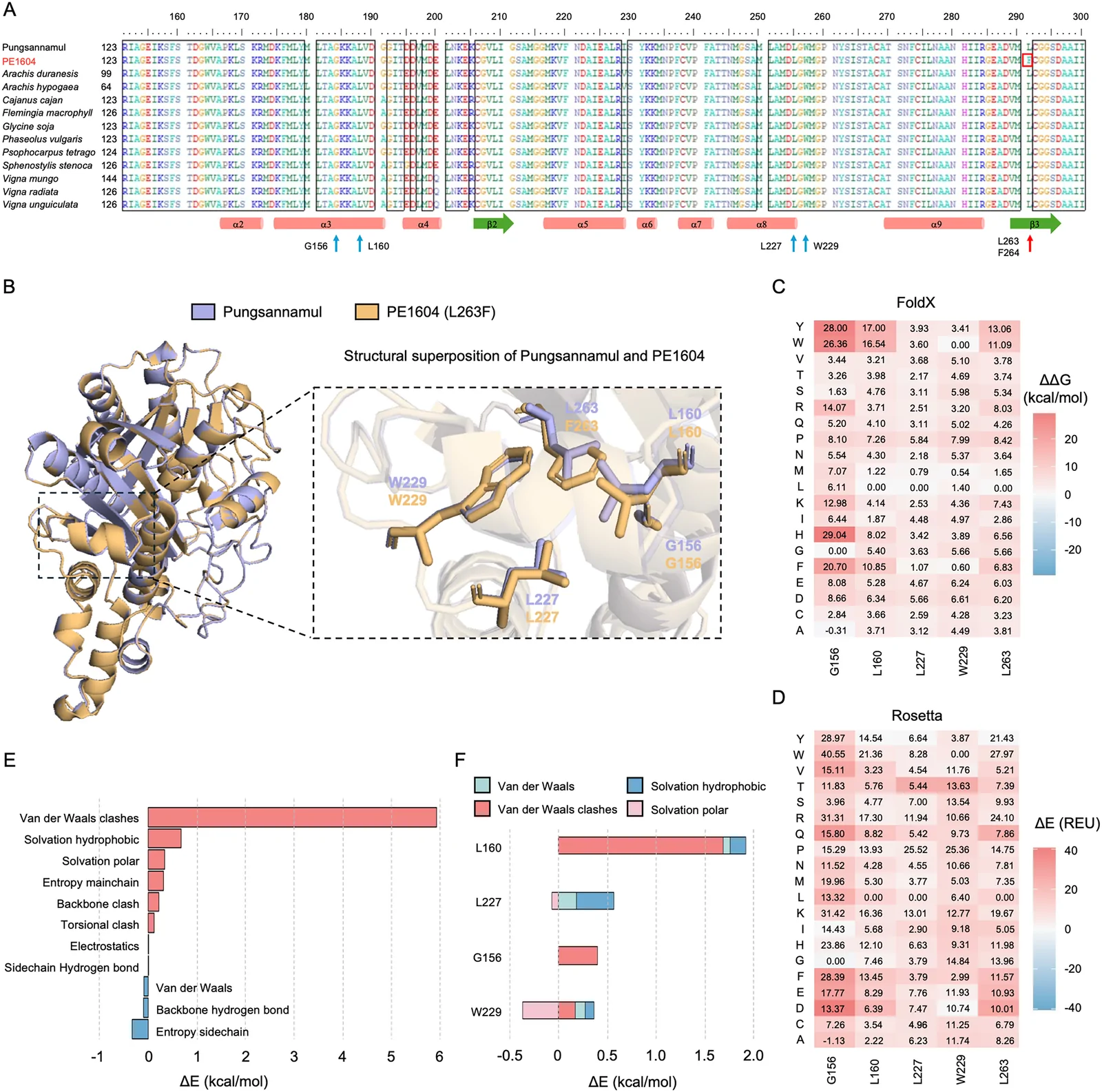
Computational analysis of the structural and energetic effects of the KASII-A^L263F^ in PE1604. **(A)** Multiple sequence alignment of KASII-A amino acid sequence from Pungsannamul and PE1604 with KASII-A homologs from 11 species of the family Fabaceae. Secondary structural elements are indicated below the alignment. The positions of Gly156, Leu160, Leu227, Trp229, and Leu263 are indicated by arrows, with the L263F amino acid substitution in PE1604 highlighted in red. **(B)** Structural superposition of KASII-A from Pungsannamul and KASII-A^L263F^ from PE1604. The enlarged view shows the local structural environment surrounding residue 263, including G156, L160, L227, and W229. **(C)** FoldX-based computational mutagenesis analysis of G156, L160, L227, W229, and L263. Heatmap values represent the predicted change in protein stability (ΔΔG, kcal/mol) resulting from substitution of each residue with the indicated amino acid. **(D)** Rosetta-based computational mutagenesis analysis of the same residues. Heatmap values represent changes in Rosetta energy (ΔE, Rosetta energy units [REU]) for the indicated amino acid substitutions. **(E)** Decomposition of the predicted energetic effect associated with the L263F amino acid substitution into individual energy terms calculated using FoldX. Values are expressed as ΔE in kcal/mol. **(F)** FoldX-based decomposition of energetic changes at residues G156, L160, L227, and W229 induced by the L263F amino acid substitution. Values are expressed as ΔE in kcal/mol.

### 3.4 Genetic validation of the KASII-A^L263F^ effect in two F_2_ populations

To determine whether the predicted structural effect of KASII-A^L263F^ is associated with the high palmitic acid phenotype of PE1604, we evaluated the phenotypic effect of the *KASII*-*A* allele in two F_2_ populations derived from Pungsannamul × PE1604 and Seonpung × PE1604. Pungsannamul and Seonpung were have distinct genetic backgrounds (**Supplementary Table 3**), whereas PE1604 was derived from Pungsannamul through EMS mutagenesis and therefore share the Pungsannamul genetic background (Chae et al., 2013). The parental lines exhibited differences in both seed size and total fatty acid content. The mean individual seed weights of Pungsannamul and PE1604 were both approximately 0.11 g, indicating that PE1604 did not differ in seed size from its original cultivars, whereas Seonpung produced substantially larger seeds with a mean weight of 0.26 g (**Figures 4A, B**). The parental lines also differed in total fatty acid content (**Figure 4C**). Pungsannamul and PE1604 contained 205.6 mg FA/g seed weight and 195.7 mg FA/g seed weight, respectively, whereas Seonpung contained 255.9 mg FA/g seed weight. These parental differences provided two contrasting populations in which to evaluate whether the effect of the *KASII*-*A* allele on palmitic acid composition was consistently expressed and how the resulting compositional changes were reflected in the amount of palmitic acid accumulated in the seed. A SimpleProbe assay targeting the C to T nucleotide substitution in *KASII*-*A* was developed to genotype the individual F_2_ seeds of the two F_2_ populations (**Supplementary Figure 2**). In the Pungsannamul × PE1604 population, 44 F_2_ seeds were *KASII*-*A*/*KASII*-*A* homozygotes, 74 were *KASII*-*A*/*kasII*-*a* heterozygotes, and 32 were *kasII*-*a*/*kasII*-*a* homozygotes. In the Seonpung × PE1604 population, the 38 F_2_ seeds were *KASII*-*A*/*KASII*-*A* homozygotes, 73 were *KASII*-*A*/*kasII*-*a* heterozygotes, and 39 were *kasII*-*a*/*kasII*-*a* homozygotes. The genotype of the F_2_ seeds of each population fitted the expected 1:2:1 segregation ratio by chi-square analysis (**Supplementary Table 4**). The composition and content of the five major fatty acids showed continuous distributions and transgressive segregation in each F_2_ population (**Supplementary Figure 9**). These distributions indicated substantial phenotypic variation within both populations, enabling evaluation of the effect of the *KASII*-*A* genotype on seed fatty acid composition and content.

**Figure 4:**
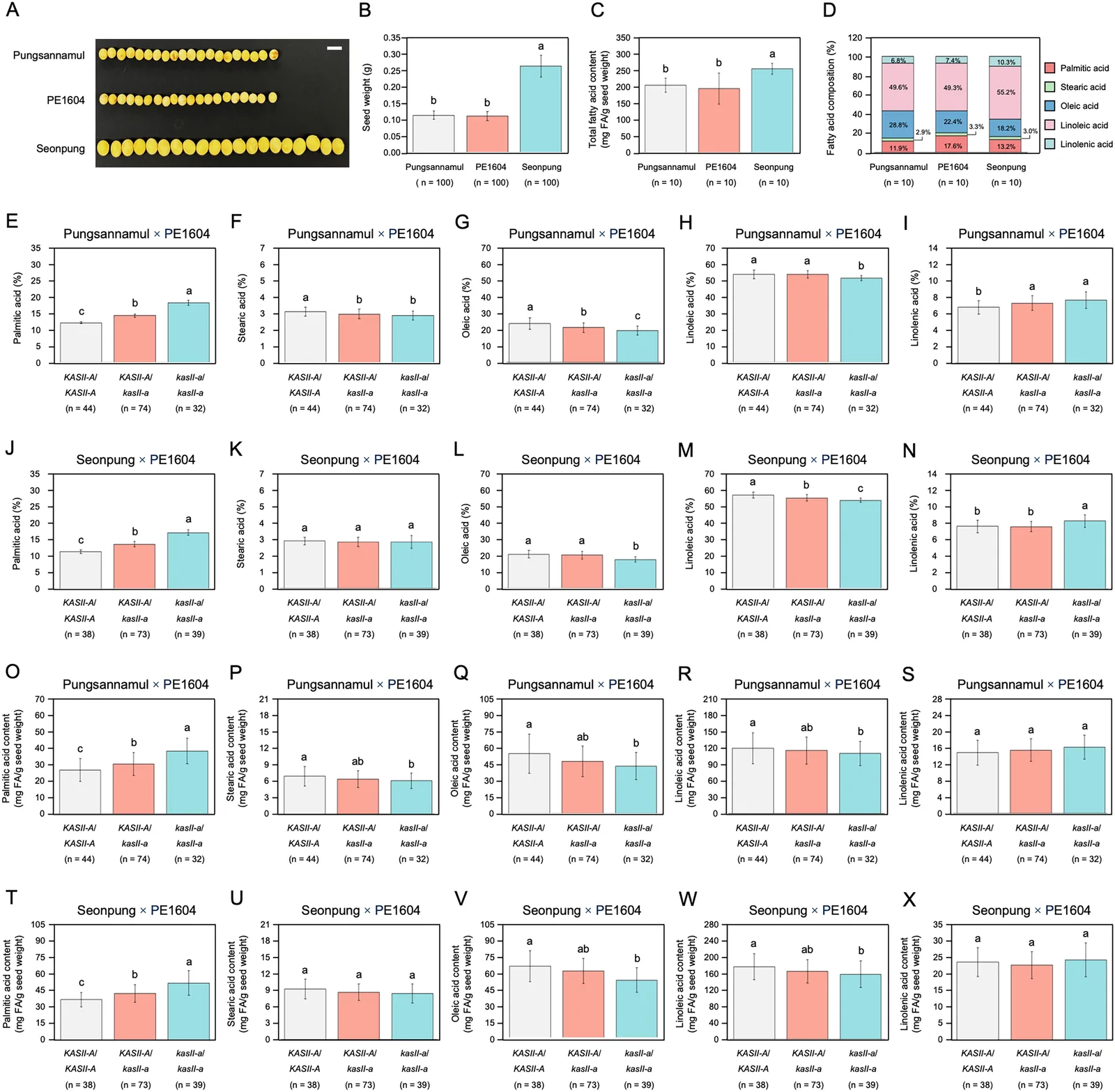
Effects of the *KASII-A* genotype on seed fatty acid composition and content in F_2_ populations derived from Pungsannamul × PE1604 and Seonpung × PE1604. **(A)** Representative seed phenotypes of Pungsannamual, PE1604, and Seonpung. Scale bar, 1 cm. **(B)** Individual seed weight of Pungsannamul, PE1604, and Seonpung (n = 100). **(C)** Total fatty acid content in seeds of Pungsannamul, PE1604, and Seonpung (n = 10). Total fatty acid content was calculated as the sum of the contents of the five major fatty acids (palmitic, stearic, oleic, linoleic, and linolenic acids). The values are expressed as mg fatty acid (FA)/g seed weight. **(D)** Fatty acid composition of seeds from Pungsannamul, PE1604, and Seonpung (n = 10). The relative proportions of palmitic, stearic, oleic, linoleic, and linolenic acids were calculated based on the total content of these five fatty major acids and expressed as percentages. **(E–I)** Seed fatty acid composition of F_2_ seeds from the Pungsannamul × PE1604 cross according to *KASII-A* genotype. **(J–N)** Seed fatty acid composition of F_2_ seeds from the Seonpung × PE1604 cross according to *KASII-A* genotype. **(O–S)** Seed fatty acid content of same Pungsannamul × PE1604 F_2_ seeds. **(T–X)** Seed fatty acid content of same Seonpung × PE1604 F_2_ seeds. Different lowercase letters indicate significant differences among the allelic combination of *KASII*-*A* as determined by one-way ANOVA followed by Tukey’s honestly significant difference (HSD) test (*p* < 0.05).

The composition of the five major fatty acids in F_2_ seed from both populations was compared based on their allelic combination of the *KASII*-*A* allele (**Figures 4E–N**). In both populations, palmitic acid composition was significantly higher in *kasII*-*a*/*kasII*-*a* homozygotes than in *KASII*-*A*/*KASII*-*A* homozygotes and *KASII*-*A*/*kasII*-*a* heterozygotes, demonstrating a consistent association between the recessive *kasII*-*a* allele and increased palmitic acid composition. In addition, homozygous recessive *kasII*-*a* allele of F_2_ seeds exhibited a lower oleic acid composition compared to another allelic combination of *KASII*-*A*. Interestingly, linoleic acid composition was significantly lower in homozygous recessive *kasII*-*a* allele of F_2_ seeds, indicating that the increased palmitic acid composition was accompanied by changes in the relative distribution of unsaturated oleic or linoleic acid composition. This pattern is consistent with previous reports showing that high palmitic acid was accompanied by reduced composition of linoleic acid (Park et al., 2016). In addition, to determine whether these compositional changes were accompanied by changes in the actual individual fatty acid content per unit seed mass, we quantified the content of each fatty acid in the same F_2_ seed (**Figures 4O–X**). Consistent with the compositional analysis, palmitic acid content was significantly higher in *kasII*-*a*/*kasII*-*a* homozygotes in both populations, while oleic and linoleic acid content was significantly reduced. These results indicate that the *kasII*-*a* allele affected both relative fatty acid composition and individual fatty acid content per unit seed mass. Taken together, these genetic results, together with the structural and computational analyses, are consistent with reduced KASII-A function associated with the PE1604 derived KASII-A^L263F^ allele and increased palmitic acid accumulation in soybean seed.

### 3.5 Breeding potential of KASII-A^L263F^ across contrasting parental lines

To investigate how parental fatty acid characterization affects the phenotypic outcome of KASII-A^L263F^, homozygous recessive *kasII*-*a* of F_2_ seed from the Pungsannamul × PE1604 and Seonpung × PE1604 crosses were directly compared with their parental lines (**Figure 5**). Palmitic acid composition was similar between the two homozygous recessive *kasII*-*a* of F_2_ seed, reaching 18.1% in the Pungsannamul × PE1604 cross and 17.4% in the Seonpung × PE1604 cross. Both values did not differ statistically from that of PE1604 (17.6%) and were significantly higher than those of Pungsannamul and Seonpung (**Figure 5A**). In contrast, the compositions of the other major fatty acids differed among the parental lines and homozygous recessive *kasII*-*a* F_2_ seed (**Figures 5B–E**). Based on these differences, we hypothesized that the palmitic acid content associated with KASII-A^L263F^ would depend on the fatty acid characteristics of the parental line. To test this hypothesis, we investigated fatty acid content to determine whether parental differences in fatty acid content were retained in homozygous recessive *kasII*-*a* F_2_ seed (**Figures 5F–J**). Palmitic acid content differed between the homozygous recessive *kasII*-*a* F_2_ seed from two crosses despite their similar palmitic acid composition. Homozygous recessive *kasII*-*a* F_2_ seed from the Pungsannamul × PE1604 cross contained 38.2 mg FA/g seed weight, whereas those from the Seonpung × PE1604 cross contained 51.9 mg FA/g seed weight (**Figure 5F**). Similar patterns were observed for the other major fatty acids. The contents of stearic, linoleic, and linolenic acids were higher in homozygous recessive *kasII*-*a* F_2_ seed from the Seonpung × PE1604 cross than in those from the Pungsannamul × PE1604 cross (**Figures 5G–J**). This difference is consistent with the higher total fatty acid content observed in the Seonpung background (**Figure 4C**). In contrast, oleic acid content did not differ significantly between the two populations. This may reflect the higher oleic acid composition of Pungsannamul and PE1604 relative to Seonpung, together with the reduction in oleic acid associated with the loss-of-function of KASII-A. These results indicate that the palmitic acid content achieved in *kasII*-*a* F_2_ seed reflects both the compositional effect of KASII-A^L263F^ and the fatty acid accumulation characteristics of the parental line. From a breeding perspective, combining KASII-A^L263F^ with parental lines showing greater fatty acid accumulation may allow the increase in palmitic acid composition to be accompanied by an increase in palmitic acid content.

**Figure 5:**
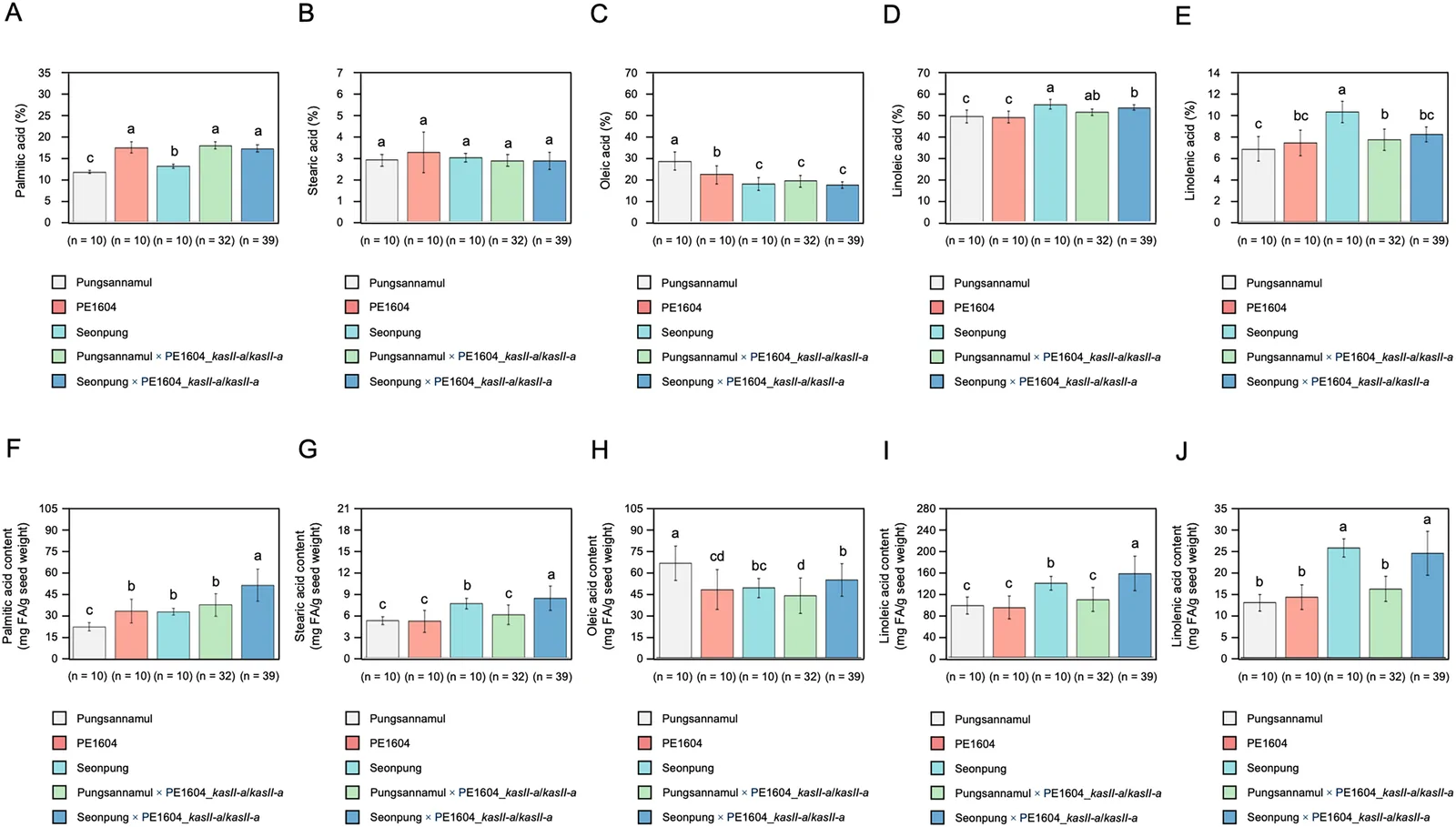
Comparison of seed fatty acid composition and content among parental lines and homozygous *kasII*-*a* F_2_ seed. **(A)** Palmitic acid composition in Pungsannamul, PE1604, Seonpung, and homozygous *kasII*-*a*/*kasII*-*a* F_2_ seed derived from the Pungsannamul × PE1604 and Seonpung × PE1604 crosses. **(B)** Stearic acid composition in the same parental lines and F_2_ seed. **(C)** Oleic acid composition in the same parental lines and F_2_ seed. **(D)** Linoleic acid composition in the same parental lines and F_2_ seed. **(E)** Linolenic acid composition in the same parental lines and F_2_ seed. **(F)** Palmitic acid content in Pungsannamul, PE1604, Seonpung, and homozygous *kasII*-*a*/*kasII*-*a* F_2_ seed derived from the two crosses. **(G)** Stearic acid content in same parental lines and F_2_ seed. **(H)** Oleic acid content in same parental lines and F_2_ seed. **(I)** Linoleic acid content in same parental lines and F_2_ seed. **(J)** Linolenic acid content in same parental lines and F_2_ seed. Different lowercase letters indicate significant differences among groups as determined by one-way ANOVA followed by Tukey’s honestly significant difference (HSD) test (*p* < 0.05).

## 4 Discussion

This study identified a novel *KASII*-*A* allele associated with elevated palmitic acid in soybean and investigated its effect from structural, computational, genetic, and breeding perspectives. Sequence analysis of PE1604 identified a C to T nucleotide substitution in *KASII*-*A* that replaces leucine with phenylalanine (**Figures 1A, B**; **Supplementary Figure 4**), although no coding-sequence polymorphism was detected in *KASII*-*B* (**Supplementary Figure 5**). Leu263 is located within a highly conserved region of KASII-A proteins and is conserved among KASII-A homologs from 11 species of the family Fabaceae (**Figure 1C**; **Supplementary Figure 7**). Structural comparison showed substitution of leucine with the bulkier aromatic phenylalanine alters residue packing around residue 263 without producing a major change in the overall KASII-A protein fold (**Figure 1D**). These observations suggested that the functional consequence of L263F may arise from a local structural effect rather than from a large-scale conformational change. We further investigated whether residue 263 is directly associated with the catalytic machinery of KASII-A. KASII catalyzes the elongation of palmitoyl-ACP to β-ketostearoyl-ACP, and impairment of this reaction provides a biochemical explanation of increased palmitic acid accumulation (**Figure 2A**). Structural comparison with *E*. *coli* FabF-C16AcpP complex showed that residue 263 is spatially separated from the catalytic residues and from the path occupied by the C16 substrate mimic in the acyl-binding region (**Figure 2E**). Also, the relative arrangement of the catalytic residues was essentially preserved in KASII-A^L263F^. L263F is unlikely to affect KASII-A through direct disruption of substrate binding or catalytic residue geometry. Previous structural studies of condensing enzymes have shown that catalytic activity depends on both precise organization of active site channel and maintenance of a tightly packed thiolase-like fold (Huang et al., 1998; Moche et al., 1999; Borgaro et al., 2011; Mindrebo et al., 2020; Huang et al., 2024). Therefore, L263F amino acid substitution may represent a different structural route to impaired *KASII*-*A* function, rather than altering residues involved directly in substrate recognition or catalysis. The computational analyses provide an alternative explanation for the functional effect of L263F (**Figure 3**). FoldX and Rosetta independently predicted an unfavorable energetic effect of the substitution (**Figures 3C, D**). Systematic substitution of Leu263 and residues located in close spatial proximity to it further showed that energetic penalties were generally lowest for side chain sizes close to those of the native residues. At residue 263, substitutions with either smaller or larger side chains than leucine were less favorable, indicating a narrow tolerance for side chain volume at this residue. This pattern is consistent with a residue whose size is closely matched to its local packing environment rather than one selected for a specific catalytic or polar interaction. Energy decomposition provided additional support for this interpretation (**Figure 3E**). Most of the unfavorable FoldX energy associated with L263F was attributable to van der Waals clashes. In addition, analysis of the energy contributions of individual residues within KASII-A carrying the L263F amino acid substitution showed that the energetic effect was not confined to Phe263 itself but extended to surrounding residues (**Figure 3F**). Leu160 showed the largest unfavorable contribution, and the distance between residue 160 and residue 263 decreased after substitution of leucine with phenylalanine. These results indicate that introduction of the larger aromatic side chain changes the local packing network within the hydrophobic core. Taken together, conservation and structural analyses, active site comparison, and computational energy calculations support a structural model in which L263F may reduce KASII-A protein stability through unfavorable local packing while leaving the overall fold and active site architecture intact. The choice of FoldX and Rosetta is supported by systematic benchmarking showing strong correspondence with experimentally measured variant effect and improved performance when the two methods are combined (Gerasimavicius et al., 2023). A relevant biochemical precedent was reported by Carlsson et al. (2002), who showed that a Leu to Phe amino acid substitution in *E. coli* KASI reduced substrate binding and condensation activity and attributed this effect to steric crowding and structural instability. However, KASII-A protein abundance and the catalytic activity of KASII-A^L263F^ were not measured directly in the present study. Therefore, the proposed destabilization mechanism should be regarded as a structure-based hypothesis consistent with, but not proven by, the genetic and phenotypic data.

The genetic analyses demonstrated that the PE1604 derived *KASII*-*A* allele was associated with the fatty acid phenotype in segregating populations (**Figure 4**). In both the Pungsannamul × PE1604 and Seonpung × PE1604 crosses, KASII-A genotypes segregated according to the expected 1:2:1 ratio (**Supplementary Table 4**). Homozygous recessive *kasII*-*a* F_2_ seed showed increased palmitic acid composition and content in both populations. The consistent association between the homozygous recessive *kasII*-*a* F_2_ seed and elevated palmitic acid in the two crosses provides genetic support for the functional effect of the PE1604 derived *KASII*-*A* allele. This effect is comparable to those observed for previously reported loss-of-function *KASII* alleles in soybean. The J10 line, which carries a 206, 203 bp-long deletion encompassing the *KASII*-*A* gene, exhibited a palmitic acid composition of 18.0%, whereas the M22 line, which encompassing the 26 bp deletion in the sixth intron of *KASII*-*B* that disrupts normal splicing, exhibited a palmitic acid composition of 16.3 (Anai et al., 2012). In addition, our previous study showed that the EMS-induced KASII-A^G309D^ allele in the PE1544 line increased the palmitic acid composition to 16.1% (Kim et al., 2025). In the present study, homozygous *kasII*-*a* F_2_ seed derived from the Pungsannamul × PE1604 and Seonpung × PE1604 crosses exhibited palmitic acid composition of 18.1% and 17.4%, respectively. These values are comparable to that observed in the J10 line carrying a complete deletion of *GmKASII*-*A*. Taken together, these results indicate that the KASII-A^L263F^ allele has a strong effect among the characterized *KASII*-*A* alleles. In addition, because this allele is caused by a single nucleotide substitution rather than a large genomic deletion, it may provide a practical advantage for breeding by minimizing undesirable genomic alterations and potential linkage drag associated with large deletion alleles. Previous studies of soybean KASII mutants have primarily characterized their phenotypic effects as changes in the relative fatty acid composition (Aghoram et al., 2006; De Vries et al., 2011; Anai et al., 2012; Head et al., 2012; Devereaux et al., 2024; Kim et al., 2025). Fatty acid composition effectively describes shifts in the relative fatty acid profile but does not indicate how much of a specific fatty acid is accumulated per unit seed mass. The content of an individual fatty acid depends on both its relative composition and the total fatty acid contents in the seed. Consequently, similar changes in fatty acid composition can result in different fatty acid contents when total fatty acid contents differ among parental lines. This distinction became particularly clear when homozygous *kasII*-*a* F_2_ seed from the two crosses were compared with their parental lines (**Figure 5**). Palmitic acid composition was similar between homozygous progeny from the Pungsannamul × PE1604 and Seonpung × PE1604 crosses and was comparable to that of PE1604 (**Figure 5A**). In contrast, the compositions of the other major fatty acids differed among the parental lines and homozygous *kasII*-*a* F_2_ seed (**Figures 5B–E**). These differences indicate that the overall fatty acid phenotype of progeny carrying KASII-A^L263F^ also reflects the fatty acid characteristics of the parental lines. A clear difference between the two crosses was observed for fatty acid content. Homozygous *kasII*-*a* F_2_ seed from the two crosses showed similar palmitic acid compositions but differed substantially in palmitic acid content (**Figure 5F**). Homozygous *kasII*-*a* F_2_ seed from Seonpung × PE1604 cross accumulated more palmitic acid per unit seed mass than those from the Pungsannamul × PE1604 cross. The contents of stearic, linoleic, and linolenic acids were also generally higher in homozygous *kasII*-*a* F_2_ seed from the Seonpung × PE1604 cross, consistent with the greater fatty acid accumulation observed in the Seonpung parental line (**Figures 5G–J**). These results indicate that the palmitic acid content achieved in homozygous *kasII*-*a* F_2_ seed reflects both the effect of KASII-A^L263F^ on fatty acid composition and the fatty acid accumulation characteristics of the parental lines. We emphasize that KASII-A^L263F^ is associated with altered fatty acid composition, consistent with reduced elongation of palmitoyl-ACP to stearoyl-ACP, whereas total fatty acid accumulation is a separate polygenic property of the genetic background. Total fatty acid accumulation is a separate polygenic property of the genetic background, and the present study does not identify the loci responsible for it. Therefore, the distinction drawn here is methodological rather than mechanistic. The amount of a target fatty acid delivered by a composition modifying allele is the product of the compositional shift that the allele causes and the accumulation capacity of the background into which it is introduced, and that product cannot be inferred from composition data alone. Our two populations show this directly, since nearly identical palmitic acid compositions of 18.1% and 17.4% corresponded to markedly different palmitic acid contents of 38.2 and 51.9 mg FA/g seed weight.

This relationship has an important implication for the use of KASII-A^L263F^ in soybean oil breeding. These results suggest that introducing KASII-A^L263F^ into soybean cultivars with greater fatty acid accumulation may increase palmitic acid content while retaining the elevated palmitic acid composition. Broadly, these results show how fatty acid composition and fatty acid accumulation can be considered together in molecular soybean breeding. KASII-A^L263F^ modifies fatty acid composition through palmitoyl-ACP to β-ketostearoyl-ACP elongation step, whereas differences among soybean cultivars contribute to variation in fatty acid accumulation. Other characterized alleles modify fatty acid composition at different steps of fatty acid metabolism. For example, *FAD2* alleles increase oleic acid by reducing its conversion to linoleic acid (Pham et al., 2010; Jo et al., 2022), whereas *SACPD*-*C* alleles increase stearic acid by reducing the conversion of stearic acid to oleic acid (Byfield et al., 2006; Zhang et al., 2008; Boersma et al., 2012; Ruddle et al., 2013; Jeong et al., 2018). Therefore, combining such fatty acid modifying alleles with cultivars showing greater fatty acid accumulation may provide a practical approach for developing soybean lines with targeted fatty acid composition and increased contents of the desired fatty acids. Two further points are relevant to the potential use of this allele in soybean breeding. PE1604 did not differ significantly from its progenitor cultivar Pungsannamul in mean individual seed weight, with both lines averaging approximately 0.11 g per seed (**Figure 4B**), or in total fatty acid content, which was 195.7 and 205.6 mg FA/g seed weight in PE1604 and Pungsannamul, respectively (**Figure 4C**). These results indicate that the L263F amino acid substitution is not associated with an obvious effect on seed size or total fatty acid accumulation in the Pungsannamul genetic background. However, other agronomic and seed quality traits, including seed protein content, plant growth habit, days to maturity, and yield-related traits, were not evaluated in the present study. Such evaluation represents an important next step in establishing the practical breeding value of KASII-A^L263F^ allele.

## 5 Conclusion

This study identified KASII-A^L263F^ as a novel soybean allele associated with increased seed palmitic acid composition. Structural and computational analyses support a mechanism in which the L263F amino acid substitution may affect KASII-A protein stability through unfavorable local packing rather than through direct alteration of the active site. In addition, genetic analyses in two F_2_ populations demonstrated an increase in palmitic acid composition in homozygous recessive *kasII*-*a* F_2_ seed. However, the palmitic acid content achieved in homozygous recessive *kasII*-*a* F_2_ seed differed according to the fatty acid accumulation characteristics of the parental lines. These findings show that the breeding value of KASII-A^L263F^ depends on both its consistent effect on palmitic acid composition and the fatty acid accumulation characteristics of the soybean cultivar into which it is introduced. Therefore, combining KASII-A^L263F^ with cultivars showing greater fatty acid accumulation may provide an effective strategy for increasing palmitic acid content while retaining the elevated palmitic acid composition conferred by the allele. PE1604 and the KASII-A^L263F^ marker assay developed in this study provide useful genetic resources for molecular breeding of soybean with modified seed fatty acid composition and content.

## Data Availability

The datasets presented in this study can be found in online repositories. The names of the repository/repositories and accession number(s) can be found in the article/**Supplementary Material**.
