# Supplementary material for: Integrated structural, computational, and genetic analyses link KASII-AL263F to increased palmitic acid composition and highlight its potential in soybean oil breeding
Source: Front Plant Sci. 2026 Oct 12;17:1983954. doi: 10.3389/fpls.2026.1983954 (PMC13696491; doi:10.3389/fpls.2026.1983954)
Supplement: Supplementary file 1 [file Table1.docx]

**Supplementary Table 1. The altered seed fatty acid profile of 163 EMS-induced mutant and two Korean soybean cultivars.**

| Accessions | Fatty acid (Mean ± SD, %) | | | | |
| --- | --- | --- | --- | --- | --- |
|  | Palmitic acid  (C16:0) | Stearic acid  (C18:0) | Oleic acid  (C18:1) | Linoleic acid  (C18:2) | Linolenic acid  (C18:3) |
| PE0075 | 9.38±0.24 | 2.86±0.12 | 33.72±1.70 | 45.01±1.12 | 9.03±0.22 |
| PE0084 | 11.50±0.07 | 3.01±0.00 | 17.17±0.07 | 56.54±0.13 | 11.79±0.57 |
| PE0156 | 9.82±0.07 | 3.04±0.05 | 34.39±0.45 | 44.20±0.19 | 8.56±0.15 |
| PE0202 | 9.68±0.15 | 2.95±0.13 | 23.56±0.49 | 51.77±0.22 | 12.03±0.56 |
| PE0204 | 9.90±0.17 | 3.26±0.06 | 34.38±0.57 | 42.96±0.10 | 9.50±0.23 |
| PE0219 | 9.70±0.12 | 3.29±0.16 | 34.64±1.28 | 44.18±0.77 | 8.19±0.23 |
| PE0222 | 9.02±0.20 | 2.29±0.10 | 15.64±0.31 | 60.00±0.37 | 13.05±0.24 |
| PE0235 | 9.34±0.03 | 2.15±0.00 | 13.56±0.23 | 59.81±0.04 | 15.14±0.16 |
| PE0239 | 10.53±0.11 | 2.84±0.03 | 32.38±0.18 | 45.21±0.03 | 9.04±0.08 |
| PE0281 | 10.16±0.04 | 3.24±0.01 | 22.29±0.21 | 48.39±0.02 | 15.92±0.17 |
| PE0287 | 10.89±0.06 | 3.61±0.06 | 20.96±0.27 | 51.97±0.20 | 12.57±0.07 |
| PE0289 | 10.36±0.09 | 3.58±0.07 | 18.94±0.17 | 52.78±0.17 | 14.34±0.15 |
| PE0293 | 9.30±0.17 | 3.48±0.02 | 34.44±0.71 | 43.95±0.42 | 8.83±0.10 |
| PE0312 | 10.40±0.12 | 3.30±0.11 | 32.24±0.07 | 45.99±0.13 | 8.07±0.03 |
| PE0328 | 10.34±0.13 | 2.94±0.08 | 18.94±0.44 | 56.08±0.15 | 11.69±0.51 |
| PE0341 | 11.39±0.07 | 3.64±0.12 | 20.91±0.57 | 52.20±0.19 | 11.86±0.63 |
| PE0361 | 13.28±0.05 | 3.77±0.01 | 20.36±0.19 | 51.26±0.00 | 11.32±0.13 |
| PE0386 | 9.94±0.07 | 3.26±0.01 | 33.76±0.09 | 44.52±0.03 | 8.52±0.04 |
| PE0388 | 13.10±0.10 | 2.90±0.02 | 16.55±0.28 | 55.04±0.05 | 12.40±0.11 |
| PE0390 | 9.37±0.02 | 3.47±0.04 | 20.80±0.53 | 54.17±0.06 | 12.19±0.53 |
| PE0422 | 9.84±0.15 | 2.73±0.02 | 16.73±0.09 | 56.83±0.31 | 13.87±0.24 |
| PE0429 | 10.25±0.08 | 2.83±0.01 | 14.40±0.00 | 59.10±0.06 | 13.42±0.04 |
| PE0438 | 9.33±0.07 | 2.96±0.03 | 13.97±0.18 | 57.03±0.09 | 16.71±0.31 |
| PE0458 | 8.70±0.17 | 2.80±0.02 | 32.78±0.83 | 41.58±0.33 | 14.14±0.31 |
| PE0478 | 8.95±0.04 | 3.61±0.01 | 32.20±0.13 | 46.55±0.04 | 8.68±0.06 |
| PE0479 | 9.92±0.22 | 3.56±0.11 | 21.77±0.91 | 52.86±0.43 | 11.90±0.69 |
| PE0529 | 8.24±0.08 | 3.26±0.06 | 47.90±0.41 | 30.24±0.13 | 10.36±0.14 |

**Supplementary Table 1. (Continued)**

| Accessions | Fatty acid (Mean ± SD, %) | | | | |
| --- | --- | --- | --- | --- | --- |
|  | Palmitic acid  (C16:0) | Stearic acid  (C18:0) | Oleic acid  (C18:1) | Linoleic acid  (C18:2) | Linolenic acid  (C18:3) |
| PE0541 | 10.68±0.08 | 3.01±0.10 | 20.62±0.33 | 53.91±0.44 | 11.78±0.65 |
| PE0576 | 9.51±0.06 | 2.61±0.06 | 33.54±0.12 | 45.19±0.16 | 9.15±0.07 |
| PE0585 | 9.20±0.09 | 2.24±0.02 | 22.00±0.25 | 53.77±0.03 | 12.80±0.16 |
| PE0606 | 9.54±0.11 | 3.09±0.08 | 34.23±0.32 | 44.22±0.34 | 8.93±0.17 |
| PE0616 | 9.90±0.09 | 3.36±0.07 | 32.13±0.09 | 46.22±0.10 | 8.38±0.02 |
| PE0638 | 10.93±0.04 | 2.93±0.03 | 18.72±1.28 | 54.64±0.81 | 12.78±0.54 |
| PE0640 | 9.51±0.25 | 3.08±0.11 | 34.12±0.80 | 45.15±0.53 | 8.15±0.09 |
| PE0715 | 9.41±0.01 | 3.13±0.03 | 32.54±0.96 | 46.08±0.88 | 8.84±0.04 |
| PE0739 | 10.30±0.08 | 3.12±0.03 | 32.15±0.05 | 46.22±0.02 | 8.21±0.04 |
| PE0743 | 9.64±0.09 | 3.40±0.09 | 34.05±0.27 | 44.64±0.49 | 8.27±0.05 |
| PE0757 | 9.82±0.05 | 3.27±0.08 | 34.63±0.51 | 44.48±0.17 | 7.80±0.21 |
| PE0793 | 10.16±0.01 | 3.36±0.15 | 31.25±0.99 | 46.65±1.10 | 8.58±0.03 |
| PE0833 | 10.26±0.02 | 3.26±0.05 | 31.09±1.04 | 46.60±1.02 | 8.79±0.09 |
| PE0838 | 9.77±0.11 | 3.04±0.08 | 33.20±0.19 | 45.02±0.18 | 8.97±0.17 |
| PE0860 | 9.40±0.15 | 3.42±0.12 | 34.04±0.14 | 45.06±0.21 | 8.08±0.11 |
| PE0863 | 9.75±0.10 | 2.90±0.11 | 33.79±0.17 | 45.11±0.11 | 8.45±0.26 |
| PE0866 | 9.40±0.11 | 3.42±0.14 | 34.04±0.33 | 45.06±0.35 | 8.08±0.01 |
| PE0867 | 9.72±0.20 | 2.81±0.07 | 36.29±0.81 | 43.52±0.82 | 7.67±0.15 |
| PE0869 | 10.00±0.28 | 3.15±0.07 | 32.42±0.50 | 45.81±0.55 | 8.62±0.26 |
| PE0875 | 9.64±0.00 | 2.78±0.13 | 32.20±0.08 | 45.85±0.29 | 9.52±0.24 |
| PE0882 | 9.79±0.18 | 2.88±0.11 | 33.37±0.05 | 45.14±0.11 | 8.82±0.23 |
| PE0884 | 9.71±0.24 | 2.85±0.10 | 32.84±0.79 | 46.21±0.94 | 8.39±0.28 |
| PE0887 | 9.63±0.12 | 2.82±0.09 | 32.93±0.11 | 46.71±0.33 | 7.91±0.24 |
| PE0888 | 9.76±0.06 | 3.14±0.16 | 32.96±0.23 | 45.63±0.18 | 8.51±0.32 |
| PE0894 | 9.76±0.18 | 2.98±0.05 | 33.90±0.38 | 45.08±0.61 | 8.27±0.36 |
| PE0899 | 9.71±0.09 | 2.87±0.03 | 32.31±0.21 | 46.44±0.25 | 8.66±0.10 |
| PE0907 | 9.94±0.07 | 2.78±0.01 | 32.57±0.14 | 45.92±0.06 | 8.78±0.26 |
| PE0908 | 8.68±0.15 | 3.08±0.08 | 44.13±1.75 | 36.38±1.70 | 7.73±0.02 |
| PE1012 | 9.55±0.20 | 2.76±0.05 | 35.51±0.11 | 42.29±0.37 | 9.89±0.11 |

**Supplementary Table 1. (Continued)**

| Accessions | Fatty acid (Mean ± SD, %) | | | | |
| --- | --- | --- | --- | --- | --- |
|  | Palmitic acid  (C16:0) | Stearic acid  (C18:0) | Oleic acid  (C18:1) | Linoleic acid  (C18:2) | Linolenic acid  (C18:3) |
| PE1028 | 10.20±0.02 | 2.83±0.09 | 33.33±0.19 | 45.55±0.42 | 8.08±0.51 |
| PE1067 | 11.86±0.23 | 2.73±0.00 | 13.35±0.31 | 56.42±0.07 | 15.66±0.16 |
| PE1077 | 11.41±0.29 | 3.52±0.10 | 33.15±0.49 | 43.67±0.18 | 8.26±0.28 |
| PE1080 | 9.40±0.21 | 3.37±0.17 | 32.32±0.21 | 46.64±0.59 | 8.27±0.01 |
| PE1101 | 10.16±0.01 | 2.90±0.12 | 31.89±0.46 | 46.59±0.41 | 8.46±0.16 |
| PE1115 | 10.23±0.07 | 3.09±0.16 | 32.13±0.44 | 45.82±0.16 | 8.74±0.38 |
| PE1117 | 10.09±0.03 | 3.12±0.10 | 33.77±0.48 | 44.63±0.27 | 8.40±0.08 |
| PE1148 | 10.08±0.05 | 3.38±0.03 | 33.08±0.22 | 44.27±0.16 | 9.19±0.03 |
| PE1149 | 10.99±0.06 | 3.17±0.01 | 33.57±0.02 | 43.94±0.11 | 8.33±0.02 |
| PE1166 | 9.94±0.03 | 3.37±0.00 | 32.51±0.11 | 45.08±0.19 | 9.10±0.05 |
| PE1172 | 9.53±0.05 | 3.13±0.10 | 32.51±0.48 | 46.49±0.13 | 8.33±0.21 |
| PE1174 | 10.33±0.08 | 2.59±0.13 | 22.05±0.99 | 52.64±0.65 | 12.38±0.28 |
| PE1198 | 10.57±0.04 | 2.77±0.01 | 18.07±0.11 | 55.80±0.05 | 12.78±0.21 |
| PE1207 | 10.16±0.04 | 3.45±0.03 | 32.43±0.21 | 45.63±0.16 | 8.33±0.01 |
| PE1260 | 9.77±0.03 | 3.08±0.06 | 32.49±0.04 | 45.93±0.02 | 8.73±0.04 |
| PE1281 | 9.49±0.18 | 3.31±0.06 | 32.73±0.73 | 45.70±0.36 | 8.76±0.13 |
| PE1319 | 10.10±0.03 | 3.41±0.08 | 35.46±0.35 | 42.78±0.24 | 8.24±0.00 |
| PE1326 | 9.92±0.02 | 2.81±0.03 | 32.18±0.14 | 44.97±0.14 | 10.12±0.06 |
| PE1350 | 9.63±0.11 | 3.08±0.01 | 32.08±0.17 | 46.71±0.06 | 8.49±0.01 |
| PE1364 | 9.91±0.11 | 3.03±0.06 | 33.81±0.77 | 44.91±0.52 | 8.33±0.08 |
| PE1366 | 9.52±0.04 | 3.06±0.10 | 33.70±0.06 | 44.88±0.03 | 8.84±0.11 |
| PE1388 | 9.72±0.08 | 2.89±0.08 | 32.82±0.09 | 45.77±0.23 | 8.80±0.16 |
| PE1437 | 10.16±0.23 | 3.02±0.03 | 31.85±0.93 | 46.12±0.75 | 8.84±0.02 |
| PE1470 | 10.04±0.03 | 2.96±0.09 | 32.41±0.01 | 45.79±0.34 | 8.81±0.20 |
| PE1482 | 9.82±0.19 | 3.22±0.18 | 33.47±0.94 | 45.18±1.03 | 8.31±0.10 |
| PE1544 | 15.44±0.27 | 3.29±0.02 | 21.69±0.42 | 49.61±0.27 | 9.96±0.14 |
| PE1569 | 11.59±0.12 | 4.01±0.05 | 16.73±0.10 | 52.86±0.08 | 14.80±0.25 |
| PE1604 | 16.14±0.02 | 4.15±0.04 | 20.43±0.09 | 49.51±0.03 | 9.77±0.03 |
| PE1610 | 10.70±0.13 | 2.96±0.05 | 18.81±0.35 | 53.19±0.07 | 14.34±0.10 |
| PE1622 | 15.22±0.44 | 3.15±0.11 | 25.13±0.27 | 46.06±0.41 | 10.44±0.41 |

**Supplementary Table 1. (Continued)**

| Accessions | Fatty acid (Mean ± SD, %) | | | | |
| --- | --- | --- | --- | --- | --- |
|  | Palmitic acid  (C16:0) | Stearic acid  (C18:0) | Oleic acid  (C18:1) | Linoleic acid  (C18:2) | Linolenic acid  (C18:3) |
| PE1663 | 9.59±0.13 | 2.86±0.06 | 20.55±1.70 | 54.36±1.53 | 12.64±0.18 |
| PE1664 | 9.92±0.14 | 2.88±0.01 | 34.43±0.00 | 44.97±0.18 | 7.81±0.03 |
| PE1669 | 10.12±0.05 | 3.20±0.04 | 33.73±0.80 | 44.58±0.87 | 8.38±0.02 |
| PE1679 | 12.86±0.38 | 3.69±0.01 | 25.67±0.13 | 47.70±0.24 | 10.07±0.02 |
| PE1683 | 10.16±0.38 | 3.06±0.15 | 19.17±1.64 | 55.20±1.51 | 12.41±0.34 |
| PE1685 | 10.87±0.07 | 2.96±0.05 | 18.00±0.04 | 55.12±0.01 | 13.05±0.05 |
| PE1696 | 9.60±0.06 | 3.39±0.01 | 32.24±0.03 | 46.22±0.11 | 8.55±0.21 |
| PE1702 | 11.11±0.18 | 2.96±0.04 | 18.75±1.63 | 52.90±0.52 | 14.28±0.98 |
| PE1740 | 10.45±0.16 | 3.53±0.06 | 31.31±0.80 | 46.05±0.82 | 8.67±0.20 |
| PE1742 | 13.46±0.10 | 3.32±0.08 | 26.39±0.02 | 46.60±0.24 | 10.23±0.43 |
| PE1757 | 10.14±0.04 | 2.89±0.04 | 20.22±4.12 | 52.67±2.10 | 14.08±2.01 |
| PE1759 | 10.02±0.43 | 3.52±.12 | 36.17±3.42 | 42.05±2.28 | 8.24±0.59 |
| PE1775 | 9.79±0.07 | 3.06±0.07 | 33.28±0.92 | 45.12±0.69 | 8.75±0.09 |
| PE1787 | 9.67±0.01 | 2.87±0.06 | 32.91±0.12 | 45.96±0.04 | 8.59±0.12 |
| PE1791 | 9.98±0.24 | 3.18±0.05 | 32.59±0.29 | 45.17±0.07 | 9.09±0.07 |
| PE1794 | 9.82±0.03 | 3.21±0.04 | 32.55±0.33 | 45.12±0.41 | 9.30±0.01 |
| PE1804 | 10.27±0.08 | 3.34±0.12 | 32.07±0.20 | 45.97±0.33 | 8.36±0.07 |
| PE1850 | 9.61±0.11 | 2.80±0.06 | 32.39±0.35 | 46.37±0.28 | 8.83±0.11 |
| PE1866 | 10.14±0.13 | 2.91±0.06 | 32.61±0.01 | 46.17±0.02 | 8.17±0.09 |
| PE1867 | 9.87±0.08 | 3.25±0.00 | 32.56±0.54 | 45.49±0.53 | 8.83±0.08 |
| PE1886 | 10.93±0.10 | 3.10±0.00 | 19.43±0.48 | 54.14±0.23 | 12.41±0.15 |
| PE1889 | 9.73±0.06 | 3.05±0.13 | 31.03±1.30 | 47.20±0.97 | 8.99±0.26 |
| PE1962 | 9.56±0.23 | 3.02±0.03 | 39.95±0.09 | 38.63±0.29 | 8.85±0.06 |
| PE1972 | 9.78±0.14 | 3.77±0.05 | 32.24±0.08 | 45.18±0.14 | 9.04±0.15 |
| PE1975 | 9.29±0.12 | 2.97±0.01 | 34.42±1.10 | 44.58±0.96 | 8.75±0.03 |
| PE1996 | 10.19±0.11 | 3.33±0.06 | 31.96±0.30 | 45.50±0.41 | 9.01±0.05 |
| PE2009 | 10.05±0.22 | 3.41±0.14 | 32.01±1.72 | 45.14±0.90 | 9.40±0.74 |
| PE2020 | 9.79±0.10 | 3.68±0.02 | 32.36±0.34 | 45.55±0.50 | 8.62±0.25 |
| PE2030 | 10.03±0.16 | 3.48±0.02 | 32.23±0.24 | 45.49±0.13 | 8.76±0.06 |

**Supplementary Table 1. (Continued)**

| Accessions | Fatty acid (Mean ± SD, %) | | | | |
| --- | --- | --- | --- | --- | --- |
|  | Palmitic acid  (C16:0) | Stearic acid  (C18:0) | Oleic acid  (C18:1) | Linoleic acid  (C18:2) | Linolenic acid  (C18:3) |
| PE2052 | 9.31±0.00 | 3.01±0.04 | 36.00±0.58 | 42.95±0.61 | 8.73±0.06 |
| PE2062 | 9.80±0.01 | 2.35±0.00 | 33.73±0.10 | 44.57±0.04 | 9.55±0.08 |
| PE2067 | 9.84±0.03 | 2.95±0.02 | 35.58±0.14 | 43.30±0.09 | 8.33±0.00 |
| PE2106 | 9.63±0.01 | 2.60±0.02 | 21.38±0.07 | 52.98±0.14 | 13.41±0.03 |
| PE2110 | 10.50±0.08 | 2.98±0.01 | 24.95±0.40 | 48.20±0.27 | 13.37±0.23 |
| PE2122 | 14.33±0.13 | 3.49±0.04 | 23.14±0.60 | 48.49±0.22 | 10.54±0.28 |
| PE2139 | 13.12±0.37 | 3.30±0.03 | 23.29±1.58 | 49.56±0.94 | 10.73±0.30 |
| PE2154 | 9.95±0.16 | 3.44±0.02 | 19.67±0.92 | 55.17±0.11 | 11.77±0.85 |
| PE2155 | 11.01±0.25 | 3.20±0.11 | 21.46±0.34 | 52.16±0.16 | 12.17±0.06 |
| PE2166 | 8.81±0.01 | 3.24±0.04 | 37.60±0.08 | 40.45±0.16 | 9.91±0.06 |
| PE2184 | 10.80±0.07 | 2.19±0.02 | 32.73±0.60 | 43.74±0.46 | 10.55±0.05 |
| PE2258 | 9.97±0.18 | 3.58±0.08 | 32.02±0.73 | 46.13±0.50 | 8.30±0.13 |
| PE2311 | 10.75±0.09 | 2.54±0.02 | 19.43±0.30 | 54.80±0.21 | 12.48±0.03 |
| PE2331 | 10.39±0.01 | 2.53±0.00 | 20.93±0.12 | 52.64±0.06 | 13.52±0.07 |
| PE2333 | 8.38±0.21 | 2.31±0.01 | 12.42±0.01 | 59.52±0.29 | 17.37±0.09 |
| PE2485 | 10.80±0.09 | 2.83±0.02 | 26.12±0.82 | 46.96±0.62 | 13.29±0.13 |
| PE2540 | 8.80±0.05 | 2.59±0.04 | 19.83±0.31 | 53.10±0.24 | 15.69±0.15 |
| PE2551 | 10.59±0.07 | 3.40±0.01 | 31.97±0.47 | 45.91±0.23 | 8.13±0.17 |
| PE2651 | 13.11±0.25 | 2.48±0.03 | 18.13±0.07 | 50.23±0.51 | 16.06±0.37 |
| PE2698 | 10.35±0.14 | 3.19±0.03 | 32.99±0.54 | 45.07±0.40 | 8.41±0.02 |
| PE2707 | 10.00±0.01 | 3.41±0.02 | 33.22±0.10 | 44.46±0.01 | 8.91±0.11 |
| PE2719 | 10.22±0.09 | 2.97±0.03 | 33.34±0.65 | 43.55±0.43 | 9.91±0.09 |
| PE2724 | 10.33±0.7 | 3.35±0.02 | 22.16±0.25 | 51.08±0.12 | 13.09±0.07 |
| PE2742 | 11.44±0.08 | 2.79±0.01 | 19.70±0.04 | 53.69±0.00 | 12.39±0.04 |
| PE2772 | 9.57±0.01 | 2.92±0.07 | 32.20±0.02 | 46.99±0.06 | 8.31±0.10 |
| PE2776 | 9.91±0.02 | 3.12±0.01 | 33.42±0.01 | 44.54±0.04 | 9.01±0.03 |
| PE2778 | 9.93±0.02 | 3.01±0.01 | 32.34±0.01 | 45.47±0.00 | 9.25±0.01 |
| PE2782 | 10.38±0.07 | 2.82±0.07 | 21.50±1.14 | 53.30±0.43 | 11.99±0.58 |
| PE2865 | 10.02±0.02 | 3.42±0.02 | 20.28±0.09 | 54.00±0.08 | 12.27±0.01 |
| PE2907 | 10.31±0.01 | 2.82±0.11 | 32.97±0.18 | 46.45±0.10 | 7.44±0.18 |

**Supplementary Table 1. (Continued)**

| Accessions | Fatty acid (Mean ± SD, %) | | | | |
| --- | --- | --- | --- | --- | --- |
|  | Palmitic acid  (C16:0) | Stearic acid  (C18:0) | Oleic acid  (C18:1) | Linoleic acid  (C18:2) | Linolenic acid  (C18:3) |
| PE2922 | 9.84±0.04 | 3.22±0.04 | 32.13±0.77 | 45.99±0.80 | 8.82±0.03 |
| PE2926 | 10.06±0.01 | 3.24±0.07 | 32.31±0.11 | 46.21±0.09 | 8.18±0.08 |
| PE2942 | 9.67±0.20 | 3.00±0.02 | 31.65±1.23 | 46.71±0.93 | 8.97±0.07 |
| PE2945 | 9.90±0.11 | 3.41±0.03 | 18.41±0.05 | 55.23±0.05 | 13.04±0.02 |
| PE2953 | 9.82±0.09 | 2.27±0.02 | 17.95±0.13 | 55.44±0.15 | 14.52±0.38 |
| PE2955 | 9.89±0.10 | 3.36±0.04 | 32.21±0.46 | 45.38±0.34 | 9.16±0.07 |
| PE2961 | 9.56±0.08 | 2.85±0.03 | 32.83±0.20 | 46.36±0.27 | 8.41±0.01 |
| PE2971 | 9.20±0.01 | 2.96±0.03 | 34.18±0.30 | 45.32±0.21 | 8.34±0.07 |
| PE2972 | 9.53±0.01 | 3.05±0.03 | 33.70±0.04 | 44.58±0.03 | 9.14±0.03 |
| PE3057 | 9.63±0.07 | 3.60±0.00 | 34.53±0.54 | 43.73±0.29 | 8.50±0.18 |
| PE3059 | 10.00±0.06 | 3.13±0.03 | 32.32±0.06 | 46.14±0.13 | 8.42±0.16 |
| PE3066 | 9.76±0.04 | 3.52±0.01 | 31.94±0.14 | 46.58±0.19 | 8.21±0.02 |
| PE3076 | 9.71±0.06 | 2.88±0.11 | 33.68±0.03 | 45.55±0.26 | 8.18±0.06 |
| PE3078 | 9.87±0.08 | 3.26±0.05 | 33.11±0.23 | 45.37±0.23 | 8.40±0.03 |
| PE3097 | 9.59±0.04 | 3.23±0.02 | 33.97±0.04 | 44.26±0.01 | 8.95±0.01 |
| PE3103 | 10.18±0.10 | 3.26±0.04 | 31.68±0.84 | 46.23±0.70 | 8.65±0.08 |
| PE3167 | 9.80±0.00 | 3.13±0.00 | 32.02±0.13 | 46.76±0.02 | 8.28±0.11 |
| PE3177 | 9.94±0.06 | 3.40±0.03 | 32.54±0.35 | 45.39±0.31 | 8.75±0.06 |
| Pungsannamul | 11.70±0.37 | 5.22±0.38 | 18.28±0.92 | 54.25±0.93 | 10.55±0.64 |
| Seonpung | 12.27±0.97 | 3.73±0.52 | 18.32±1.92 | 55.94±1.31 | 9.73±0.58 |

**Supplementary Table 2. Primers used for Sanger sequencing**

| Primer name | Forward (5’ **→** 3’) | Reverse (5’ **→** 3’) |
| --- | --- | --- |
| *KASII*-*A*-1 | CGTCACTTCACTTTGATGTGTCTCG | TGCCTATCATTCACAAGAGTAAATG |
| *KASII*-*A*-2 | GTGCACCTTTAGGATTATGGTTCCTTG | GACAAAATGAGAGTATCATAAACCAACTTTTG |
| *KASII*-*A*-3 | GCTTTGTTATGTGCTCTTTGGATATTAG | CAAAAGTGATAGATGCATTTAATTTTGTGATG |
| *KASII*-*A*-4 | CAGCCTGTTAGGCTTGTTCTTAAAAC | GGCACAAAGCTATATTCTAAACAAAGCAC |
| *KASII*-*A*-5 | GACGGCCAATAACCCTTGTCTTC | CCACCTCCCTTTTAACTGATATATACC |
| *KASII*-*B*-1 | CACTCGCTCGCAACCACAA | CACAATTCCAAAGGCATAGATCAGC |
| *KASII*-*B*-2 | GTGGTAGACATTTACTTCCATGAATGATTG | CACCAATGATTGCTGGATTAATATTGTATTC |
| *KASII*-*B*-3 | GGTGTTAACAAATTTAGTAAACATGAAATTTG | CAAAGGTAATTGACACAGTTACAAACG |
| *KASII*-*B*-4 | CTCCCAATCTTTATTTGATGTGCATATCATC | GGAATCATAATAACATTTCAATTGGCTAGTAAG |
| *KASII*-*B*-5 | CATGACAATTCCGGTATAGTGAAAGAG | CATTGTCTGGGTACTCAGTACCTGC |

**Supplementary Table 3. Pedigree and breeding background of the soybean cultivars used as parental lines**

| Cultivars | Pedigree | Year of release | Seed size | Seed weight (g/100 seeds) | Seed coat color | Flower color | Reference |
| --- | --- | --- | --- | --- | --- | --- | --- |
| Pungsannamul | Pangsa × KLS87092 | 1996 | Small | 11.3 | Yellow | Purple | Lee et al., 2015 |
| Seonpung | Suwon 224 × YS1325-3S-2 | 2013 | Large | 25.9 | Yellow | White | Lee et al., 2015; Kim et al., 2017 |

**Supplementary Table 4. Segregation analysis of the *KASII*-*A* allele in F_2_ populations derived from Pungsannamul × PE1604 and Seonpung × PE1604 crosses.**

| Population | Progeny | Observed for segregation ratio | | | Expected for segregation ratio | | | Chi-Square  (1:2:1) | *p* |
| --- | --- | --- | --- | --- | --- | --- | --- | --- | --- |
|  |  | *KASII*-*A*/  *KASII*-*A* | *KASII*-*A*/  *kasII*-*a* | *kasII*-*a/*  *kasII*-*a* | *KASII*-*A*/  *KASII*-*A* | *KASII*-*A*/  *kasII*-*a* | *kasII*-*a/*  *kasII*-*a* |  |  |
| Pungsannamul × PE1604 | F_2_ (n = 150) | 44 | 74 | 32 | 37.5 | 75 | 37.5 | 1.95 | 0.38 |
| Seonpung × PE1604 | F_2_ (n = 150) | 38 | 73 | 39 | 37.5 | 75 | 37.5 | 0.12 | 0.94 |

n, Number of plants.
